# Cocreating First Steps, a Toolkit to Improve Adolescent Sexual and Reproductive Health Services: Qualitative Human-Centered Design Study With Hispanic and Black Adolescent Mothers in New York City

**DOI:** 10.2196/60692

**Published:** 2024-11-19

**Authors:** Lauren Gerchow, Yzette Lanier, Anne-Laure Fayard, Allison Squires

**Affiliations:** 1 Rory Meyers College of Nursing New York University New York, NY United States; 2 Nova School of Business and Economics Carcavelos Portugal

**Keywords:** adolescent, reproductive health, sexual health, cocreation, co-design, human-centered design

## Abstract

**Background:**

Adolescent voices are frequently excluded from sexual and reproductive health (SRH) research. Despite progressive policies and access to SRH care, adolescents in New York City who live in neighborhoods with high poverty and those who identify as Black or Hispanic experience poor SRH outcomes, including high rates of unplanned pregnancies and sexually transmitted infections.

**Objective:**

This qualitative study aims to guide Black and Hispanic adolescent mothers in identifying problem areas in SRH care and cocreate health service recommendations with input from health care stakeholders to address those problems and improve SRH experiences.

**Methods:**

Through ethnographic interview methods, adolescent mothers in New York City shared their experiences from before pregnancy through parenting and identified problem areas in adolescent SRH services and education. Data were analyzed inductively and using situational analysis. Adolescent participants attended 2 cocreation workshops. In the first workshop, they confirmed interview findings, set priorities, and created rough prototypes. Following the first workshop, health care providers were interviewed to inform refinement of the rough prototypes. Adolescents further developed prototypes in the second cocreation workshop and named the resulting toolkit.

**Results:**

A total of 16 adolescent mothers participated in 47 interviews, and 10 (63%) participants attended at least 1 cocreation workshop. They highlighted deficiencies in sexual health education and emphasized the roles of health care providers and parents, rather than schools, in improving it. Adolescent participants designed recommendations for adolescents and health care providers to support quality conversations between adolescents, parents, and health care providers and created a preappointment checklist to help young patients initiate conversations with health care providers. Young participants stressed that sex education should address topics beyond sexually transmitted infections and pregnancy, such as emotional health and relationships. They created guidelines for health care providers outlining communication strategies to provide respectful, unbiased care and contraceptive counseling that encourages adolescent autonomy. Participants shared specific suggestions for how to support young parents respectfully. Health care stakeholders recommended adding information on confidential care; supporting lesbian, gay, bisexual, transgender, and queer youth; and focusing on improving communication between health care providers and patients rather than creating educational materials. In the second workshop, adolescent participants revised the prototypes based on feedback from health care stakeholders and named the toolkit of recommendations First Steps.

**Conclusions:**

This study highlighted the important roles that parents and health care workers play in adolescent sexual health education. Cocreated toolkits offer a practical approach for health care providers to engage adolescents and their parents in meaningful, adolescent-centered conversations that can promote health, safety, and well-being.

## Introduction

### Background

Much of the research on adolescent sexual and reproductive health (SRH) is deficit-focused, aiming to change individual behaviors. This deficit model, which focuses on challenges, rather than strengths, can perpetuate beliefs that certain groups, particularly young people of color and those living in marginalized communities, need to be rescued or saved by members of the oppressing community [1,2]. Such practices minimize the lived experiences and expertise of youth and can perpetuate cycles of poor health outcomes [1], by failing to acknowledge the root causes of health inequities, such as racism, segregation, and inequitable health policies [1,2].

Adolescent participation in SRH research is vital, given the rising rates of sexually transmitted infections (STIs) [3] and the significant racial, ethnic, geographic, and socioeconomic disparities among pregnant and parenting adolescents [4]. However, few research studies include adolescents in roles that harness their wisdom about their SRH needs and experiences. There is a need for strengths-based research approaches that engage adolescents as experts to not only improve SRH outcomes but also to address the root causes of the sustained and widening inequities.

Adolescent SRH outcomes in New York City (NYC) expose stark health inequities. Youth living in NYC neighborhoods experiencing high poverty and those who identify as Black or Hispanic experience the highest rates of pregnancy, childbirth, and STIs [5,6]. In NYC, non-Hispanic Black adolescents report pregnancies at rates 4 times higher than non-Hispanic White adolescents and 7 times higher than Asian adolescents [6]. Similar disparities are present in birth data, with Hispanic or Latinx adolescents giving birth at a rate 4 times higher than Asian adolescents and 3 times greater than non-Hispanic White adolescents.

Adolescent childbirth and parenting are associated with poor social, educational, and health outcomes for young parents and their children. Pregnant adolescents are at high risk of complications such as pre-eclampsia and labor and delivery concerns, including hemorrhage requiring blood transfusion [7]. Infants born to adolescent parents are more likely to have low Apgar scores and require assisted ventilation and intensive care [7]. Parenting adolescents have lower chances of graduating from high school and report high periods of joblessness [8,9].

Adolescents are generally at high risk of STIs, and social determinants of health including poverty and discrimination increase this risk for marginalized adolescents [10,11]. In NYC, Black adolescents and those living in neighborhoods with high poverty experience chlamydia and gonorrhea infections at the highest rates [5]. STIs are often undetected due to low rates of testing and many sexual partners experiencing asymptomatic infections [10]. Data on STI testing prevalence among adolescents in NYC is limited; however, nationally, just 20.4% of sexually active adolescents reported being tested in the last 12 months [11].

STIs are associated with adverse outcomes, including cancer, pelvic inflammatory disease, and infertility, and many STIs require lifelong treatment [12]. Female adolescents are at a higher risk for certain infections such as chlamydia related to normal biological changes associated with development [13]. Pregnant and parenting adolescents are at higher risks of STIs [13], as they are less likely to consistently use STI prevention behaviors, such as condom use [14,15].

Adolescent SRH research in the United States has focused on increasing knowledge about sexual health [16,17], increasing contraceptive use [18-21], and developing decision support tools to guide contraceptive method choice [22-24]. Many studies have found modest or no effects on sexual health outcomes. As the adolescent pregnancy rate decreases, disparities persist, and those experiencing pregnancy are more likely to develop an STI, indicating a significant gap in SRH education and health care.

Few studies have investigated adolescent family planning experiences or behaviors beyond encouraging the consistent and correct use of a contraceptive method, and even fewer have investigated this phenomenon in parenting adolescents who are at an increased risk for repeat pregnancies and STIs [25,26]. This approach to mitigating adolescent pregnancy is situated in a research approach where health care providers and researchers design models of care to address predetermined problems based on their expertise [27] and measure success using outcomes such as contraceptive uptake or STI rate. Research approaches that de-emphasize factors beyond these clinical outcomes, such as satisfaction with care, are of particular concern for adolescents who will experience age-appropriate life course changes that can affect their SRH decisions and preferences over time.

The studies that have investigated adolescent SRH with a more flexible and creative approach found that adolescent SRH decisions are dynamic and influenced by numerous factors and relationships, beyond the evidence-based effectiveness focus of providers [28-30]. For instance, one randomized controlled trial found that individually tailored motivational interviewing that allowed for flexible conversations about concerns beyond contraception, between participants and nurse interventionists, significantly reduced the rate of repeat adolescent pregnancy [31]. Adolescents are capable of describing their distinct health care needs, but they often receive care in health service settings designed for adults or preadolescent children [32]. Their voices are often not included in health services design due to barriers to their participation in research [33].

### Aims

There is a need to expand on the studies that support adolescent-centered health services and to use research approaches that are inclusive, collaborative, strengths-based, and creative to facilitate adolescent participation in the design of their SRH care. To meet this need and address the wide and sustained SRH inequities, this study aimed to (1) use ethnographic and design research to guide adolescent mothers to identify problem areas in SRH care and (2) cocreate recommendations to improve SRH education and services with both health care stakeholders and adolescent parents to improve SRH experiences. Adolescent mothers are an ideal population to participate in cocreation to improve SRH for the broader adolescent population based on their experiences with the SRH care and education systems at key time points before, during, and after an adolescent pregnancy.

## Methods

### Overview

This study presents findings from a qualitative human-centered design (HCD) study with a sample of adolescent mothers living in NYC. HCD is a research methodology in health care that considers the expertise of stakeholders, and more specifically beneficiaries of health services, essential to the problem-solving and solution-generating processes [34]. HCD uses iterative processes where researchers are deeply immersed in a specific context and take a facilitator’s role to support all stakeholders, whose expertise is recognized as central, in defining and reframing problems as well as creating and refining solutions [35-37]. HCD is an appropriate method to investigate persistent problems and disparities that are not improved by existing interventions [27]. It is a promising approach to addressing health disparities where the needs of vulnerable and marginalized populations may not be met by traditional research approaches [38].

**Figure 1:**
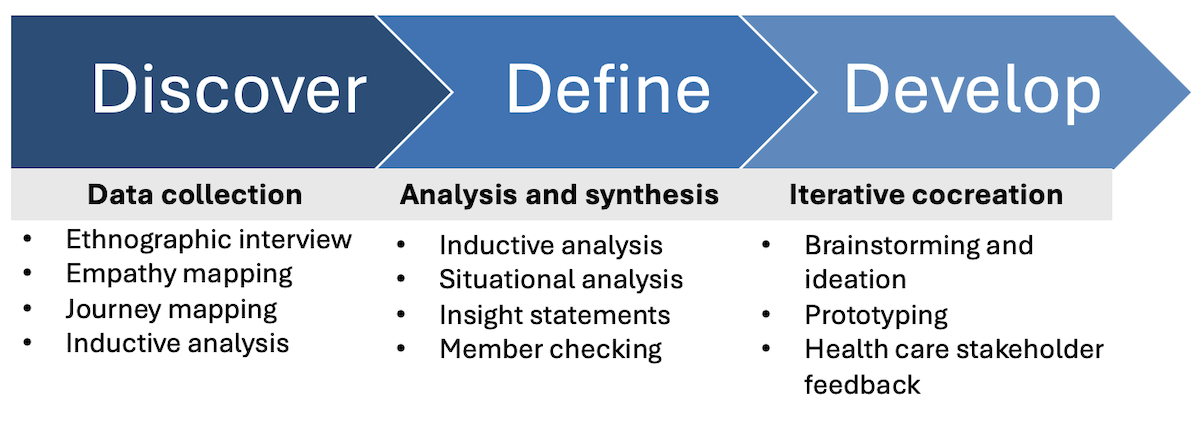
Process diagram.

HCD follows a phased process where researchers and stakeholders first investigate and define problems before they prototype and evaluate solutions. The Double Diamond, more recently called the Framework for Innovation, is a visual representation of the iterative processes used in HCD. Problem-solving and innovation in the Double Diamond framework are divided into 4 phases: discover, define, develop, and deliver [39]. In the discover phase, researchers and designers use ethnographic methods to develop a deep understanding of the experiences and contexts of service users. In the define phase, a problem is defined and redefined based on data from the discover phase and input from stakeholder groups. In the develop phase, prototypes are created to address the problems identified in the define phase and feedback is elicited to improve the prototypes. In the deliver phase, the final product is put into practice and evaluated for its impact on outcomes. This paper reports on this project’s discover, define, and develop phases. Figure 1 displays a process diagram that shows the methods of data collection, analysis, and cocreation in each of the 3 phases.

### Positionality

The first author (LG) conducted all study procedures as part of a doctoral dissertation. She is a cisgender White woman who is bilingual (Spanish and English), bicultural by heritage (Hispanic Costa Rican and White Italian heritage), and works as a maternal–child home visiting nurse. Coauthors served as mentors with expertise in health services research (AS), adolescent sexual health (YL), and HCD research (ALF).

### Theoretical Framework

HCD elevates the voices of stakeholders if carried out in its ideal, participatory form where studies acknowledge the context, culture, and political nature of complex problems. Fayard and Fathallah [40] suggested that researchers need to apply a critical stance to recognize the expertise of stakeholders, consistently reflect on positionality and the researcher’s influence on the study, and maintain a commitment to a political viewpoint that reflects the goals and needs of the participants and their communities.

To adopt this critical stance, we used intersectional feminist theory to guide data collection, analysis, and researcher reflexivity. Intersectional feminism considers the intensified effects of overlapping racism, classism, and other forms of oppression on marginalized women [41-43]. This framework encouraged continual consideration of the power matrix that adolescent parents in NYC face, including the health and socioeconomic disparities sustained by racism, poverty, and societal judgment.

### Participants and Recruitment

We recruited adolescent participants from a home visiting program for first-time parents, from a childcare program located in NYC public schools, and through snowball sampling. Participants were eligible if they were aged <20 years, lived in NYC, had English or Spanish proficiency, and had ever experienced a live birth. We recruited health care stakeholders for the develop phase by contacting members of an adolescent health special interest group of a professional organization focused on SRH and a nursing health services research collaborative. Health care stakeholders were eligible if they were licensed registered nurses (RNs), physicians, nurse practitioners, or physician assistants. We expanded inclusion criteria to include students in a degree-granting program for these health care roles to include the views and opinions of trainees with recent experiences in health education. We continued recruitment efforts until meaning saturation was achieved per Hennink et al [44], where authors developed an understanding of the various dimensions of single codes and an overall understanding of the data in both Spanish and English.

### Ethical Considerations

LG translated consent forms and all study materials into Spanish, and a native speaker confirmed translations. All study procedures and materials in English and Spanish were approved by the New York University institutional review board (FY2023-7308). New York State Public Health law considers parents aged <18 years to be adults and allows them to make health decisions for themselves and their children without parental consent. Consistent with this law, all participants, regardless of age, provided written informed consent. The consent form included specific language about the mandated reporting of suspected child abuse and neglect and the procedures to do so. In addition, participants were made aware that the research team could provide them with resources for physical health; mental health; and educational, parenting, and other needs.

All participants were aware that their participation was voluntary and could be revoked at any point during the study, and declining would not affect their relationship with their referral source. Participants received US $30 cash or gift card incentive for completing each interview, up to 3 total. Before cocreation sessions, participants were reminded of the group-based nature of the workshops and that they did not need to share their names or other identifying information with the group and could keep their cameras off. Adolescent participants received a US $50 cash or gift card incentive for cocreation workshop, up to 2 workshops.

### Discover Phase

#### Overview

The discover phase used multiple qualitative data collection tools to support immersion into the adolescent context and to build rapport and trust with participants. LG conducted all data collection following a 3-section semistructured interview guide, composed of ethnographic interview questions (section 1), card sorting (sections 1 and 2), empathy mapping (section 3), and journey mapping (section 3). Participants could elect to complete data collection in up to 3 research encounters to offer flexibility for scheduling. All participants chose the location of interviews, including an online option on Zoom (Zoom Video Communication, Inc). Participants completed a demographic survey and reported contraceptive history.

#### Ethnographic Interview

Ethnographic interviews are appropriate data collection techniques during the discover phase of HCD studies as they are exploratory and conversational and build trust with participants [45]. We used both descriptive and structural ethnographic interview questions to encourage participants to use their own language and describe how they organized their thinking about sex education, family planning, and sexual activity. The interview guide included specific questions about birth control and contraceptive counseling at time points from before their pregnancies through parenting.

#### Card Sorting

Design researchers use card sorting to build rapport through interactive activities that can ease participant discomfort in discussing sensitive topics such as sex [46,47]. Card sorting helps researchers understand and visualize how and why participants organize or categorize knowledge related to a phenomenon. In card sorting, we asked participants to group people in their lives who either should or should not speak to adolescents about sexual health and to rank the trustworthiness of sexual health information sources.

#### Mapping

Design researchers use empathy mapping [48] to uncover a person’s unspoken beliefs by dividing a page into 4 sections: do, say, think, and feel. Participants completed 4 total maps, 2 from their perspectives as adolescents and 2 regarding how they believe adults would fill out maps about adolescents. In total, 2 maps focused on adolescents generally and 2 focused on parenting adolescents. They completed each map by answering the questions, (1) “What do adolescents/parenting adolescents do/say/think/feel about sex and family planning? and (2) “What do adults do/say/think/feel about adolescent/parenting adolescent sex and family planning?”

Journey mapping [48] displays the steps people take and the emotions accompanying those actions when engaging with a service. Participants reflected on experiences receiving sex and family planning counseling before, during, and after pregnancy and described positive or negative moments along the journey. Templates and examples of completed card sorts, empathy maps, and journey maps are available upon request.

#### Data Analysis

Data analysis co-occurred with data collection. Data were analyzed inductively, first through open coding and later using focused coding to categorize findings [49]. Situational analysis derived from grounded theory [50] identified influential people, objects, and organizations that affect adolescent sexual decision-making. Findings specific to situational analysis are published in a separate manuscript [51]. Findings were synthesized into insight statements or short statements that capture the important motivations, tensions, and perspectives that encompass an HCD problem [52]. Interviews were audio recorded on a password-protected recorder, anonymized, and professionally transcribed. Audio recordings were deleted after transcription was completed and transcripts were confirmed for accuracy. Transcripts were read multiple times for immersion into the data and were analyzed using Atlas TI (ATLAS.ti Scientific Software Development GmbH). Spanish language transcripts were coded in Spanish. Analyses were confirmed through team-based discussions and through member checking, where participants offered feedback or confirmed the researcher’s findings. Reflexivity and reflection on positionality were achieved through memoing and by discussing with research mentors (AS, YL, and ALF), with the first author paying specific attention to emotional and physical responses during interviews and analyses.

### Define Phase

#### Overview

In the define phase of an HCD study, a problem is defined and redefined based on data from the discover phase and inputs from stakeholder groups. We invited participants with English proficiency who completed at least 1 interview segment to attend the first group cocreation workshop conducted on Zoom to maximize young participants’ ability to attend. Spanish-speaking workshops will be conducted in a future study, given limited resources and a smaller sample.

#### Workshop 1: Define

LG started the first workshop by providing a positionality slide and facilitating a group discussion on rules for participation, including agreeing to maintaining confidentiality, respecting others’ comments, and being open to learning new things. The first half of workshop 1 focused on supporting adolescent participants to define the key problems in SRH care and prioritize which problems to address through cocreation.

As the first step to defining the problems, LG presented insight statements. Participants had opportunities to share feedback on insight statements and suggest changes to statements if they disagreed. Together, adolescents and LG transformed insight statements into design opportunities or short statements that summarized the challenges and encouraged participants to think broadly about solutions.

### Develop Phase

In the develop phase, researchers and stakeholders create and refine solutions to design opportunities. The research team selected a toolkit to design multiple solutions that could be tailored to a specific context.

#### Workshop 1: Develop

Using a virtual whiteboard and guided by the design opportunities, the group individually and then collectively brainstormed a list of important content areas a messaging toolkit for health care providers and adolescents should address. LG encouraged participants to think about general sexual health and education and address specific contraceptive counseling needs as identified in insight statements. A second whiteboard was created considering how participants imagined the toolkit recommendations could be used. After brainstorming a range of ideas, LG presented 2 personas and accompanying scenarios for the development of rough toolkit prototypes. We created personas or fictional characters that represented different service users’ needs, experiences, pain points, and goals. LG refined rough prototypes after the first workshop based on the group’s goals and feedback during workshop discussions.

#### Health Care Professionals’ Feedback

LG conducted interviews using a semistructured interview guide to elicit feedback on prototypes developed with adolescents participating in workshop 1 with health care professionals, including RNs, pediatricians, and obstetrician-gynecologists. In addition to these interviews, LG led a group discussion in a graduate-level nursing SRH course. LG provided a summary of the findings from the discover and define phases and introduced different versions of the prototypes from the first workshop. The interview guide asked health care professionals to share feedback on the content of the prototype; the strengths, weaknesses, and usefulness of the prototype in their practice; and how they imagined the toolkit could be disseminated and used in the future. Consistent with the develop phase, versions of the toolkit were created and refined according to providers’ feedback to be shared with adolescent stakeholders in a subsequent workshop.

#### Workshop 2

In workshop 2, LG summarized health care stakeholders’ feedback on the toolkit for the adolescent participants. Discussions focused on reviewing feedback on the proposed length, toolkit content, and the primary audience. During the group discussion, LG and participants continued to iterate on and refine the prototypes. The final 15 minutes of the workshop were spent naming the toolkit, brainstorming ways to use the toolkit, and discussing future directions for the project.

## Results

### Overview

A total of 16 adolescent participants signed written informed consent. Demographics are presented in Table 1.

**Table 1:** Interview and cocreation workshop participant characteristics (N=16).

Participant characteristics			Participants, n (%)
**Age (y)**			
	14	1 (6)	
	15	1 (6)	
	16	1 (6)	
	17	5 (31)	
	18	5 (31)	
	19	3 (19)	
**Race or ethnicity**			
	Black	3 (19)	
	Both Black and Hispanic	4 (25)	
	Hispanic	9 (56)	
**Interview language**			
	English	12 (75)	
	Spanish	4 (25)	
**Highest education completed**			
	<9th grade	2 (12)	
	9th grade	4 (25)	
	10th grade	3 (19)	
	11th grade	5 (31)	
	High school diploma	1 (6)	
	High school equivalency	1 (6)	
**Birthplace**			
	United States	12 (75)	
	Dominican Republic	2 (12)	
	Ecuador	2 (12)	
**Years living in the United States for foreign-born individuals**			
	1	1 (6)	
	2-5	2 (12)	
	>5	1 (6)	
**Neighborhood income below the federal poverty level (%)**			
	<20	1 (6)	
	20-24.9	8 (50)	
	25-29.9	6 (38)	
	>30	1 (6)	
**Insurance**			
	Medicaid	13 (81)	
	Private insurance	2 (12)	
	Uninsured	1 (6)	
**Contraceptive history**			
	Condoms	12 (75)	
	Withdrawal	10 (63)	
	Natural family planning or fertility awareness	9 (56)	
	Oral contraceptive pill	6 (38)	
	Implant	6 (38)	
	Injection	4 (25)	
	Patch	3 (19)	
	Ring	0 (0)	
	Intrauterine device	0 (0)	
	Emergency contraception	3 (19)	

All participants identified as cisgender women or girls with a median age of 17.5 (IQR 17-18) years. Most participants were interviewed in English (12/16, 75%), insured by Medicaid (13/16, 81%), and had not received a high-school diploma (14/16, 88%). Participants identified as Black (3/16, 19%), both Black and Hispanic (4/16, 25%), or Hispanic (9/16, 56%). Contraceptive histories varied, with most participants having used condoms (12/16, 75%). The implant (6/16, 38%) and oral contraceptive (6/16, 38%) were the most common hormonal methods. In total, 10 (63%) of the 16 participants attended at least 1 cocreation workshop, with 9 (56%) attending each workshop and 8 (50%) attending both workshops.

### Discover Phase

#### Overview

Participants completed 47 (98%) of 48 interview segments, with 11 (69%) of the 16 participants having at least 1 in-person interview that was conducted at their homes, schools, or a community organization. The remaining interviews took place on Zoom where mapping exercises were completed using Google Slides. The interview segment duration ranged from 22 to 63 minutes. We saw no differences in interview quality by length, language, age of participant, or virtual versus in-person setting.

#### Sex Education Needs

Participants described lacking quality sex education at school, in their homes, and with health care providers. Despite living in a large city with progressive reproductive health policies around contraception and abortion, most participants received no sex education in their school or received a single class period as part of a health class that addressed other topics such as nutrition and substance use. At the same time, many shared that school is not the best place for young people to learn about sex due to the group setting and concerns that school staff do not have the training and resources necessary to provide up-to-date information.

In card sorting, all participants agreed that parents or other trusted adults in a young person’s life such as an older cousin or aunt should be speaking to adolescents about sexual health. However, in their experiences, participants said that most adults overreacted or were judgmental and uncomfortable in these conversations. Participants saw parental disapproval of sexual activity as a universal experience for all young people. In the interviews, they reflected on their experiences and wished that their parents had understood normative adolescent development, which could have created a safe environment for conversations about sexual activity and safety. On the other hand, some participants grew up in families where adolescent sex or pregnancy was seen as acceptable or inevitable, which changed the way sexual health was discussed in their homes.

In addition to parents, all participants believed that health care professionals should be educating young people about sex because of their education and training in the health care space. Some believed that health care workers could bridge the SRH communication gap between parents and their children. Many shared that pediatricians did not assess for sexual activity or did so briefly only to move on from the topic if a young patient said they were not sexually active. Instead, participants shared that they began to learn about sexual health in more detail after they discovered their pregnancy and that at this time point, they found health care providers to be judgmental and paternalistic, especially around birth control. Almost all participants experienced coercive contraceptive counseling, with providers pressuring them to long-acting reversible contraception (LARC) and providing biased counseling that did not address the side effects. Providers with the LARC-first mentality dismissed participants’ desires to use other less effective methods, discouraged LARC removal, and diminished participants’ trust.

#### Contraceptive Use

Many participants shared that they did not want to use contraception, even when actively avoiding pregnancy. Discussions about side effects took place in interviews with every participant. Many had personally experienced negative side effects, including mood changes, weight gain, and undesired vaginal bleeding or menstrual changes. Some participants described feeling uncomfortable with the idea of LARC methods being placed inside their bodies and disliking needles as reasons to decline the injection. While most participants had used condoms at least once, many described them as difficult to use consistently because of issues with partners, skin irritation, and not having or using them in the moment of sexual activity.

### Define Phase Workshop 1

During the first cocreation workshop, LG shared findings from interviews with participants in the form of insight statements and asked them for feedback and validation. Insight statements and quotes from interviews that represent each statement are provided as a Multimedia Appendix 1.

Insight statements focused on desiring adults, especially parents, to understand and accept normative adolescent sexual activity, recognizing insufficient sex education in school, and requesting that parents and health care professionals take on roles to educate adolescents about sex. Three specific insight statements guided cocreation activities, as follows: (1) young people do not have a way to learn about sex and contraception that includes their parents (or an adult they trust) and health care providers (who have the knowledge and training), (2) young people who do not want to become pregnant do not use birth control; and (3) health care providers put pressure on young people, especially young parents, to use birth control, and it affects how they counsel their patients. They do not accept “no” as an answer the first time. Using “how might we” questions, LG transformed insight statements into design opportunities to guide cocreation.

### Develop Phase

#### Workshop 1: Develop (Brainstorming Toolkit Content)

**Figure 2:**
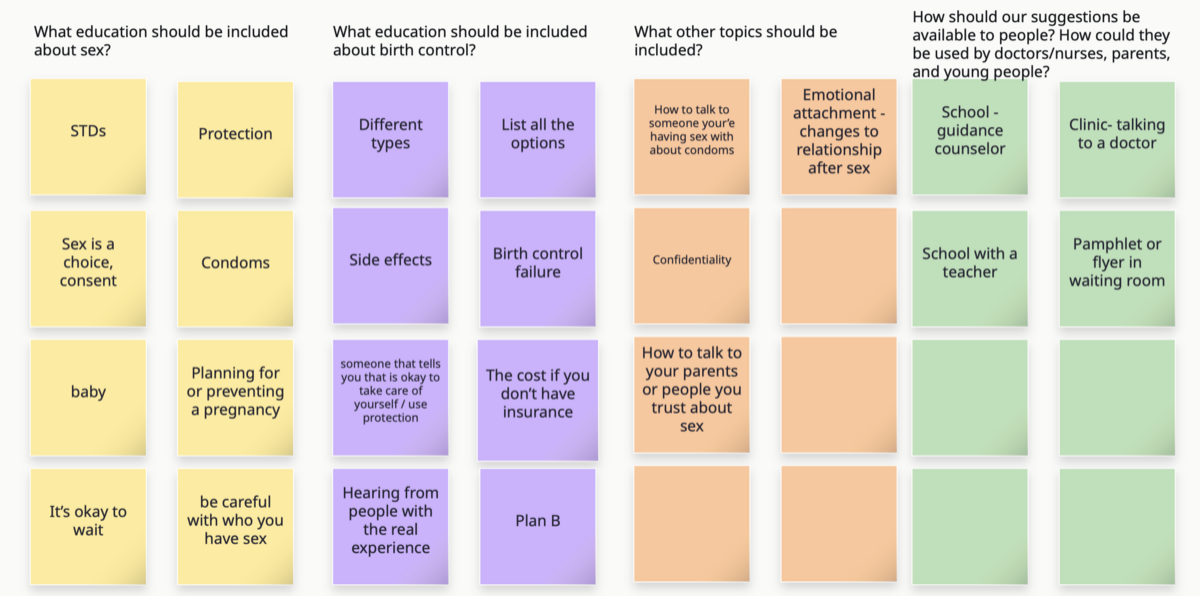
Workshop 1 content brainstorming categorized. Formatting and spelling in the figure represent the tool as it was created during the session. STD: sexually transmitted disease.

In workshop 1, the group discussed the goal of creating recommendations to help adolescents, parents, and health care providers have high-quality conversations about sexual health and contraception. The first cocreation activity asked participants to consider what SRH content should be included in the recommendations. Using sticky notes on a web-based whiteboard (Figure 2), participants brainstormed the topics they felt were important to include in sex education that would be used by adolescents, health care professionals, parents, and trusted caregivers. After initial brainstorming, the group categorized and grouped sticky notes into 3 content areas: contraceptive-specific content, general sex education, and other.

To address general sexual health issues, participants wanted content that addressed STIs, condoms, planning for or preventing pregnancy, choosing an intimate partner, and consent. Participants shared important topics specific to contraception, including a list of all the birth control options available and recognition of the birth control failure rate. Participants asked for the content to address emergency contraception specifically. Along with a complete list of all contraceptive options, participants wanted honest descriptions of side effects and desired testimonies of real people’s positive and negative experiences using a specific method. Workshop participants discussed the importance of having an adult who will not judge a young patient who wants to use contraception.

In addition to general sexual health and contraception content, participants asked for specific information related to emotional health, especially around how the relationship with a partner can change after having sex. They suggested 2 content areas about communication, including tips for how to talk to a partner about condom use and ways to talk to parents or other trusted adults about sex.

#### Workshop 1: Personas and Prototyping

LG created 2 personas (Multimedia Appendix 2) based on interview data to reflect participants’ challenges and experiences. One persona was a sexually inexperienced cisgender girl aged 14 years, Alyssia, who was in a new relationship and considering sexual activity with a new partner aged 15 years. She had a close relationship with her mother, but when they talked about sex, her mother used generic messages such as “If you do that, be safe.” She had 2 goals, first to learn about sex from her pediatrician whom she trusts and second to open a communication channel with her mother to be able to talk about these topics without her mother becoming angry or uncomfortable. At a recent routine pediatric screening, her pediatrician did not mention sexual health and she was too shy to ask for sex education.

Workshop participants brainstormed recommendations and advice to share with Alyssia and her pediatrician to help Alyssia meet her goals. Participants believed that it was the pediatrician’s job to conduct a sexual health assessment and to present sexual health as a normal conversation point. They also stressed that even if a pediatrician was using the “right words” that support normative sexual activity to introduce sexual health, young patients would need to feel that the checkup was in a safe and judgment-free environment. Participants introduced the idea of a preappointment checklist as a way for Alyssia to alert the pediatrician about sensitive topics that she wanted to discuss. They felt that this would be a way to ensure the pediatrician made time for sexual health discussions or could connect Alyssia with someone else at the clinic to continue the conversation if they ran out of time. They suggested that the checklist should include physical sexual health topics such as STIs and birth control, along with relationship and emotional health content. Participants brainstormed ways for Alyssia to have a productive appointment with her pediatrician. First, they stressed the importance of Alyssia’s pediatrician being responsible for starting the conversation about sex. Second, they shared that private conversations and an understanding of confidential care with the option to include a parent or trusted adult are the best ways for a young person to feel comfortable.

The second persona was Vanessa, a cisgender girl aged 17 years. She was a sexually active patient presenting to the gynecologist to start birth control. She expressed a desire to use the oral contraceptive pill, but the gynecologist disagreed and focused counseling on the intrauterine device and the implant. Vanessa wanted to learn more about the pill and avoid pregnancy. Workshop discussions about Vanessa’s case focused on designing recommendations for health care providers to improve contraceptive counseling experiences.

First, participants discussed what guidelines the gynecologist could follow to improve this encounter. They shared that health care providers need to be accepting of a patient’s requests while also doing their job using questions such as, “What makes you interested in the pill?” They suggested that health care providers need to begin contraceptive counseling sessions with broad and open-ended questions such as “What birth control methods did you have in mind?” rather than assuming that a young patient wants a long-acting method. They emphasized the importance of Vanessa feeling safe during the appointment and feeling comfortable speaking about her SRH needs.

The group responded to a final set of questions about how health care providers should speak to and support young parents. In terms of contraceptive counseling, participants wanted to be treated the same as adult pregnant or parenting patients and given respect and autonomy. Many shared that they wanted health care providers to care about them as a person and a parent, rather than a young person at risk of a subsequent poor outcome. Others shared that the assumption that a young mother is sexually active and desires birth control or will not be able to abstain from sexual activity is harmful and leads to a loss of trust in the health care provider.

Following this workshop, LG organized prototyped recommendations into separate “recommendations toolkits” for adolescents and health care workers. LG created an electronic mock-up of rough prototyped toolkits using the Canva software (Canva Pty Ltd).

#### Health Care Stakeholder Feedback

Prototype feedback interviews ranged from 20 to 40 minutes with 10 health care professionals, including RNs (n=6, 60%), pediatricians (n=3, 30%), and obstetricians-gynecologists (n=1, 10%). Nurses in a graduate-level SRH class in NYC (8/10 students, 80%) also provided feedback. While most (7/10, 70%) RN and obstetric stakeholders had practice experience in the NYC area, pediatric providers represented more geographic diversity, with experiences from New England, the southeastern United States, and the West Coast represented. When reviewing the prototypes, they believed that the content requested by the adolescent participants was appropriate and could help health care providers understand adolescents’ needs. Health care stakeholders were unsurprised that young parents felt judged and had negative experiences with SRH providers. To address these negative experiences, they suggested that the toolkit could focus on improving adolescent-health care provider communication, rather than duplicating preexisting educational resources. However, health care provider participants shared 1 concern about adding to the cognitive burden placed on primary care providers to serve as sex educators, given their limited time and resources.

They recommended adding information on the ability to provide confidential SRH care to minors and information on inclusive and respectful care of sexual and gender-diverse youth, including normalizing the use of pronouns. Pediatricians suggested adding practical tips for health care stakeholders to conduct routine sexual health appointments with adolescents, such as adding conversation starters or reviewing the steps of a sexual health assessment. They believed that the previsit checklist could be helpful, both for young patients and health care providers to prioritize sensitive topics and to consider including or excluding parents or supportive adults based on a young patient’s request. LG iterated on the prototype versions after interviews to present to participants in workshop 2 for additional revisions.

#### Workshop 2

In the second workshop, adolescent participants discussed health care stakeholders’ suggestions to improve communication between health care workers and adolescents, rather than recreating educational materials. Participants agreed, and cocreation workshop 2 focused on refining the toolkit made for health care providers.

First, LG shared health care stakeholders’ questions about how to help young patients feel safe to have sexual health discussions with them. Participants shared that the most important aspect of a conversation was that it was nonjudgmental and that health care providers addressed them with respect by giving them time and attention, not following checklists, or being focused on the computer. Participants shared that hearing personal stories from a health care provider about their own life or the experiences of their patients or friends can help a person to feel comfortable and humanize SRH education and counseling. Participants shared that many young patients do not know that they can receive SRH care confidentially and that this may affect their trust in a health care provider.

Participants discussed the proposed preappointment checklist and felt that it could encourage autonomy by motivating adolescents to set appointment goals and priorities. They also responded positively to the checklist having talking points related to specific SRH topics. The group reviewed the list of topics on the checklist, and participants opted to add consent and sexual violence aspects, which were not included in the sex education they had received at school or home. Finally, the group brainstormed a list of appointment “dos and don’ts” to provide concrete recommendations to guide health care providers to find the words needed to conduct sexual health assessments.

At the end of the workshop, participants took time to name the toolkit. The group agreed on First Steps as they felt that the guidelines that they had created were building blocks for health care providers to improve their SRH care to adolescents. Following the workshop, LG continued to refine the toolkit using the Canva design software and reviewed the toolkit with coauthors while considering plans for future refinement, dissemination, and implementation.

#### Final Toolkit

**Figure 3:**
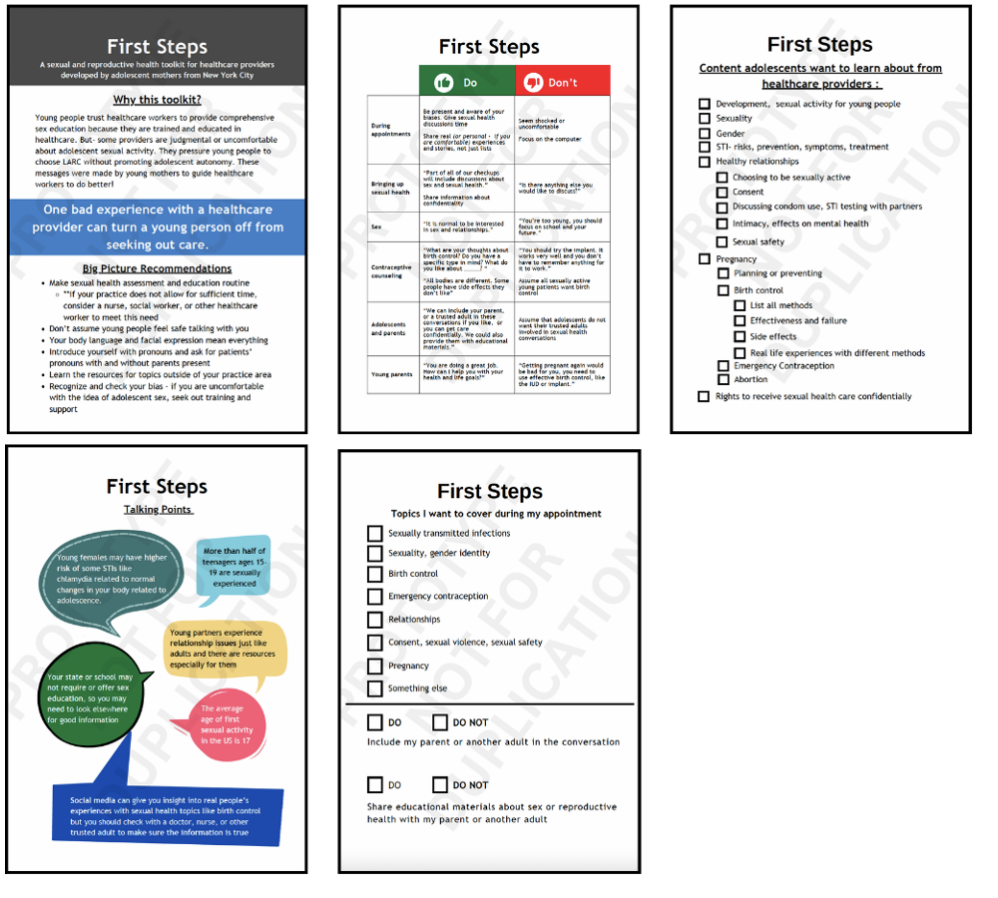
Toolkit final prototype.

The final toolkit is a 5-page document (Figure 3), with each page functioning as a stand-alone sheet that could inform care. The first page introduces the creation of the toolkit with adolescent mothers and their motivation to participate in the project based on their prior experiences with poor care. This first page offers big-picture recommendations for health care providers working with adolescent patients, including making sexual health assessment routine, recognizing biases, and considering body language and facial expression as components of a safe space.

The second page displays a table with the “dos and don’ts” for various categories, including introducing sex, contraceptive counseling, and working with young parents. Dos include offering both confidential private care and the inclusion of parents or trusted adults at appointments, sharing real people’s experiences, including their own experiences, and providing contraceptive counseling that supports adolescent autonomy. Don’ts address coercive contraceptive counseling approaches, shaming young patients for being sexually active, and assuming that all adolescent participants would like their SRH care to be kept private from their parents.

The third page offers talking points that can help normalize sexual health behavior and offer educational information about various factors that affect adolescents’ decisions and behaviors. The fourth page presents a list of SRH topics that adolescent participants wanted health care workers to be able to provide education or resources about. The preappointment checklist is the toolkit’s final page. The checklist includes a list of topics adolescents can use to alert health care providers of the SRH content they want to learn about and includes options to request the involvement of a parent or trusted adult either during an appointment or through educational materials.

## Discussion

### Principal Findings

This study used HCD and cocreation techniques with adolescent mothers and health care stakeholders to design a toolkit to help health care providers deliver high-quality, adolescent-centered SRH care. Using HCD supported us in acknowledging the importance of adolescents’ agency and recognizing their expertise as necessary components to develop health services that are meaningful and useful. Using HCD not only guided adolescent participants to identify the SRH problems they have seen and experienced but also generate solutions that are relevant to their experiences and needs [53]. While still subject to bias and power dynamics, HCD and other participatory approaches can address common concerns in research where interventions designed by experts in a field can lead to the development of culturally unsafe interventions that are ineffective or even harmful [54].

### Comparison With Prior Work

Our findings are consistent with previous literature, and through the HCD approach, these findings supported the cocreation of the First Steps toolkit. Participants described insufficient SRH education, and many believed that they did not have the knowledge they needed when they were contemplating engaging in sexual activity or discovered that they were pregnant. Similar to our participants, the SRH education that adolescents received depends on the norms and values in their homes and schools, with some favoring comprehensive sex education and others endorsing abstinence-only education or avoiding the topic [55-58].

In cocreation workshops, adolescents brainstormed ways to fill the gaps left by insufficient sex education. While participants described parents as responsible for communicating with their children about SRH, they acknowledged that some parents may not have the comfort or knowledge to do so. Instead, they identified health care workers as responsible adults with the knowledge and training to educate them, and potentially their parents, on SRH. At the same time, participants shared that their experiences with health care workers were judgmental or coercive, putting pressure on them to use a specific birth control method and focusing on risk rather than their overall health or well-being. These findings are consistent with literature examining adolescent experiences with health care providers where young patients have experienced or feared the health care provider’s judgment about their sexual health behaviors [59] or counseling that was biased due to their age [60]. Researchers have begun to recognize contraceptive coercion at the hands of health care providers, realizing that counseling methods such as tiered-effectiveness counseling are at odds with reproductive justice tenets [61]. First Steps not only confirmed these findings but also offered suggestions and actionable practice changes to address longstanding reproductive health concerns and amplified the importance of reproductive autonomy.

Toolkit recommendations focused on content areas that adolescents believed should be included in SRH education, guidance to health care workers on how to discuss sex with young patients, and a previsit checklist that could help adolescent patients alert health care staff that there are specific SRH topics they would like to discuss at a checkup. Many of these concepts are not new to the SRH literature, including respecting adolescent patients as capable of making autonomous decisions [62,63] and the importance of trust and nonjudgmental care [63,64]. However, the First Steps toolkit provides tangible recommendations for health care providers to improve health service delivery created by adolescents in their language and based on their needs and expertise. Beyond modules or training, the toolkit offers an actionable change to practice that can be implemented in diverse contexts. The toolkit can be paired with the few existing education-focused interventions that support the engagement of both parents and health care providers, such as Families Talking Together, a sex education program for Black and Latino youth aged 10 to 14 years [65].

First Steps addresses adolescent–health care provider communication, filling a gap in research where studies typically focus on increasing SRH knowledge, exploring experiences, or testing interventions designed by health care providers and researchers as the experts. First Steps, on the other hand, is innovative having been created through the phases of HCD with both adolescents and health care stakeholders, focusing on improving SRH service experiences and adolescent communication with trusted adults. The toolkit enables health care providers and researchers to use existing education resources while strengthening their relationships with adolescents and the trusted adults in their lives.

### Strengths and Limitations

This study engaged an underresearched population, adolescent mothers, to share their experiences and participate in redesigning SRH service delivery through cocreation. The use of HCD methods allowed for considerable time in the field and immersion into the adolescent context, while the workshops brought participants together to share their experiences and expertise. This study’s most significant strengths are cocreation and the design of a tangible deliverable that uses adolescent mothers’ words to recommend actionable SRH practice and education changes. Adolescent mothers are frequently overlooked in research; however, our findings, participant retention, and the toolkit deliverable collectively exemplify their wisdom and commitment to the health and well-being of their communities.

Varied data collection techniques, member checking, and additional confirmation of insight statements during group sessions strengthened the credibility of the findings. LG is an experienced home visitor with >10 years of experience working with young parents and their infants and toddlers. However, she was the sole researcher conducting interviews and facilitating cocreation workshops, which may have led to biases in analyses and interpretations. This bias was mitigated as much as possible through group-based discussions, reflection through memoing, and discussion of LG’s positionality with participants.

The adolescent participants are reflective of the adolescent parenting population in NYC in terms of race, age, and ethnicity; however, their experiences and recommendations to improve practice may not meet the needs of nonparenting adolescents, those from rural communities, or those with different heritage backgrounds, potentially limiting the transferability of the findings. The geographic diversity of health care stakeholders and the participatory HCD approach may have helped to mitigate the effect of this limitation. Similarly, all participants identified as cisgender women or girls, and their SRH experiences and proposed care solutions may not meet the needs of sexual and gender-diverse adolescents. Owing to resource constraints, LG did not recruit enough Spanish-speaking participants to conduct separate cocreation workshops with those participants; however, their perspectives from interviews were included in cocreation discussions and generally matched the perspectives of English-speaking participants.

### Implications

Methods such as HCD can guide researchers to conduct studies that support adolescents in identifying problems and creating solutions. Elevating the voices and expertise of adolescent mothers addresses a gap in research, where adolescents who are already parents are under-studied in favor of prevention programs focused on nulliparous adolescents. This study provides methodological guidance to conduct a multilingual HCD study that includes virtual and in-person interviews and a cocreation component with adolescents. Future research can support adolescents, including those who are already parents, to design sexual health education and assessments that are adolescent-centered and foster a sense of safety. Replication of the earliest phases of the project will ensure that toolkit recommendations meet the needs of diverse populations, including adolescents from rural communities, immigrants from regions and countries not represented in this study, and sexual and gender-diverse adolescents.

Health care providers can use the toolkit and introduce the preappointment checklist to their patients to normalize sexual health discussions, support parent involvement if desired, and situate themselves as sexual health educators. Additional research to further refine the prototype and consider how to best evaluate the toolkit in practice is needed and planned for future study. Health care providers working in geographically diverse settings, particularly those in states that restricted access to SRH services and education, will have unique and important insights that can inform the creation of multiple versions of the toolkit for use in diverse health services contexts, including school-based health centers.

There are opportunities for continuing education and interdisciplinary collaboration in pediatric care settings. Such practices support specialization in the various SRH topics that adolescents described as important to their overall SRH knowledge besides STIs and pregnancy, including relationship and emotional health. Adolescent health care sites can consider adding mental health providers, RNs, or other health care workers to deliver holistic SRH care and education without adding additional practice burdens on primary care providers.

### Conclusions

Despite decades of commitment from researchers, policy makers, and health care providers seeking to increase adolescent SRH knowledge and improve access to services, poor outcomes still persist and health disparities continue to widen. There is a need to address adolescent SRH concerns from the perspectives of adolescents themselves and to value and recognize their lived experiences as essential to the development of health services. This HCD study exemplifies adolescents’ wisdom, creativity, and abilities to be the designers of their own SRH care. The study supports adolescent participation in the redesign of health services and education in the United States and offers health care stakeholders the opportunity to reflect on the care they provide, with actionable and constructive practice changes that can improve SRH education, service experiences, and ultimately, outcomes.

## Appendix

Multimedia Appendix 1 Insight statements and corresponding quotes.

Multimedia Appendix 2 Personas and scenarios.

## Abbreviations

HCD: human-centered design
LARC: long-acting reversible contraception
NYC: New York City
RN: registered nurse
SRH: sexual and reproductive health
STI: sexually transmitted infection
