# Supplementary material for: Cocreating First Steps, a Toolkit to Improve Adolescent Sexual and Reproductive Health Services: Qualitative Human-Centered Design Study With Hispanic and Black Adolescent Mothers in New York City
Source: JMIR Pediatr Parent. 2024 Nov 19;7:e60692. doi: 10.2196/60692 (PMC11615555; doi:10.2196/60692)
Supplement: Multimedia Appendix 1 [file pediatrics_v7i1e60692_app1.docx]

**Multimedia Appendix 1.** Insight statements and corresponding quotes.

| Insight statement and design opportunity | Illustrative quotes |
| --- | --- |
| 1. Adults might not think it is okay, but it is normal for young people to have sex. | 1. “I think almost every single one disapproves of it. I don’t think they accept that their child is a person, and as a teenager, you’re going to do things that teenagers do, with or without your parents’ permission, with or without the knowledge that you need.” [Hispanic participant aged 19 years] 2. “They wouldn’t talk to me about that stuff, because they probably thought that I wasn’t the type to do any of that stuff.” [Hispanic participant aged 18 years] 3. “I think some people overreact. Meaning beat they kid’s ass, ground them, punish them, spread their business, embarrass them on social media. They end up leaving, harming they self, thinking of a way out, running away.” [Black and Hispanic participant aged 19 years] 4. “I think they think that young people don’t do anything. They think either they’re not going to be sexually active, they won’t be sexually active, they’re going to wait.” [Hispanic participant aged 19 years] |
| 1. Sex education is not good enough. School is not always the best spot to learn about this because it is not private, and other kids are not mature. | 1. “They was trying to skip two parts of the story. I feel like they want us to learn on our own…They were too uncomfortable to tell.” [Black and Hispanic participant aged 18 years] 2. “Oh, they had [sex ed], but I never got a schedule for that class.” [Hispanic participant aged 18 years] 3. “Yes, they don’t have that class anymore. I know that the older kids who graduated, they used to have it. We don’t have it no more. The teacher left.” [Black and Hispanic participant aged 19 years] 4. “I’m not a person to talk about that conversation around the crowded class. I like it private so I can…Because let’s say the teacher says, ‘What do you think about it?’ I won’t like others to hear what my opinion is. So yeah, I think it should be something private where you take time with your students to talk about it. But not all students will feel comfortable by saying their opinion around others.” [Hispanic participant aged 16 years] 5. “I think it was in my sophomore year of high school. Had a health education. They were talking about that. But I feel like the only reason why they were talking about that’s because I got pregnant. When I got pregnant, they started talking about sex education. Because before they would only talk about drugs and stuff like that, but after a while they started talking about how to prevent pregnancy.” [Hispanic participant aged 18 years] 6. “One of my friends today, they just brung it up to me, brung up sex ed to me today. He brung a bunch of condoms and he started throwing condoms at people, talking about, ‘No more babies.’ He a goofball, so he’s saying it and throwing condom at people. He’s like, ‘No more kids, no more babies, because I’m not having no more after this one. I’m strapping up today.’ I’m like, ‘Yo.’ He has a kid on the way. He has a girl pregnant right now.” [Black participant aged 18 years] |
| - Parents (or an adult you trust) and physicians/nurses should be talking to young people about sex. | - Parents: - “Parents for sure should…because they gave birth to me. They should tell me everything. They need to teach me everything.” [Hispanic participant aged 17 years] - “Even if they’re strict, I feel like they should know that kids will be kids and that they shouldn’t be afraid of it. So they should just give them the education, because you never know, even if you think that they’re innocent.” [Hispanic participant aged 18 years] - ​​“I'm going to say nobody [should talk to adolescents about sex] because it’s nobody business. But if it’s really, really something that you need to know, I say parents. And that’s it. Because the parent needs to know what’s going on with their kids as well.” [Black and Hispanic participant aged 18 years] - Health care professionals: - “The school counselor, the social worker, they probably would look stuff up online and then tell you about it, or they would give you advice to their best knowledge. But [my home visiting nurse] on the other hand, she actually got educated about it, knows about it, and she deals with it on a daily basis. Talking about it, she’ll answer all your questions, you’ll understand and figure out what you need to do.” [Black and Hispanic participant aged 16 years] - “Doctors definitely, because they went to school for that too. I know a lot of doctors went to school for stuff like that…But I feel only the doctors that really tells you or really sit there and listen ‘cause some doctors will sit there and listen and then take it and write it up or make a case about it or something.” [Black and Hispanic individual aged 18 years] - “Los doctores con mucha más experiencia te pueden explicar de cómo vas a experimentar eso, qué es lo que va a ocurrir en tu cuerpo, cuáles serían las consecuencias de haber hecho eso, por aconsejarte que sea lo mejor” - English translation: “Doctors with more experience can explain about how you will experience something, what is going to happen in your body, what the consequences to having done that, being able to give you advice about what would be best.” [Hispanic participant aged 14 years] |
| - Young people do not use birth control due to the following reasons: - It will not happen to me (I will not become pregnant) - I do not want my parents to find out - Side effects - Too complicated - Condom issues (eg, skin irritation, do not have them at the moment, using them ruins the moment, and not using them right) | ● “I didn’t want to do [the implant] when I gave birth because I was in so much pain. I had so much other stuff to worry about. And I had got so many needles already, so I really wasn’t trying to add that on my list.” [Black participant aged 15 years]  ● “I was like I got God on my side, for real. No, I don’t know what it is. I think it’s the-- I don’t know. I think it’s like the risk that gives us the thrill. Yes, because we’re young and dumb and want to have fun.” [Black and Hispanic participant aged 19 years]  ● “I have strict parents, so I did not want them to find out that [I was having sex] if I had a stick in the bathroom or anything.” [Hispanic participant aged 18 years]  ● “They don’t want to go through the process of putting it on and all that stuff. Or let's say that the pills don’t work and they’re like, ‘Oh, I don’t want to eat that. I don’t want to consume that,’ or like, ‘I don’t want to put none of these things on.’ Everything’s a process, so going to the doctor and putting that on, it’s a whole big process.” [Hispanic participant aged 16 years]  ● “Oh, they’re probably like, ‘Oh, I don’t like the condom, doesn’t feel the same,’ or whatever. It’s always something.” [Hispanic participant aged 16 years]  ● “Ellos sienten que van a ser juzgados. Van a ser juzgados o sienten que pueden quedar embarazadas y la van a avergonzar por eso, o rechazar por eso. Yo tengo una amiga así, que ella cambió de número de su pediatra para que la mamá no supiera, por miedo.”  ○ English translation: “They feel like they are going to be judged. They’re going to be judged or feel like that can get pregnant and they will shame her for that or reject her for that. I have a friend like that, she changed her pediatrician’s number so that her mom wouldn’t know, because of her fear.” [Hispanic participant aged 17 years] |
| - Health service needs: - Some physicians are very pushy about using birth control especially if you are a young parent. - Some physicians do not explain things in a way that is easy to understand. - Some physicians do not share the negatives about birth control. | ● “After I had my second, the midwife was telling me that I should get an IUD and stuff like that because it could get really bad if I have another baby right away. And I had told her I didn’t want the IUD and stuff. And she kept on trying to persuade me into an IUD and stuff and telling me like, ‘We could do it right now if you want us to. We're already here, we could just insert it.’ And I was telling her, ‘No, I don’t want the IUD, I don’t want it.’ And she kept on proceeding to ask me to the point where even my doula was kind of getting upset because I had already said no about three times. And then eventually she backed off and was like, ‘Okay, but we want to tell you, inform you that if you have another baby, you got a lot of complications and stuff.’ And I was like, ‘I’m aware of everything.’ They were literally massaging my stomach still, to get it out, and she was telling me about the IUD…They had to check my cervix to make sure I didn't rip. While they had the whole little rag inside, she was still asking me about the IUD, while I was in pain from a rag inside of me.” [Hispanic participant aged 19 years]  ● “The side effects. To be honest, they really don’t tell you the side effects. They don’t tell you, they’re not being honest with you, that’s what I feel. So I’d rather just not get it because I feel like the doctors would just tell you the benefits of it. They’re not going to tell you the side effects.” [Hispanic participant aged 18 years]  ● “No. No one had told me [the side effects]. The doctors, they didn’t know. They’d just be like, ‘You should use this one. It’s the best one,’ whatever. But they wouldn’t say, ‘Oh, the pills do this, do that.’ They didn't say that.” [Hispanic participant aged 16 years]  ● “I feel like doctors, school nurse, I’m in the middle because sometimes they don’t explain things good. Then they put that pressure, especially to little kids. Not little kids, but probably from 12 and up. I don’t think that's good for their mental health or something. They overthink it. They’re not explaining it in a 12-year-old mindset.” [Hispanic participant aged 18 years]  ● “So, that, I was bleeding for six months. I was bleeding for six months. Then I was bleeding for four months. Basically, I was bleeding all that time until my son was a good one years old…when I used to go to the doctor, they used to say, ‘Yeah, this is a cause of birth control.’ What you mean? I went in there. I was like, ‘Excuse me, I've been bleeding for 90 days.’ I was legit bleeding for 90 days. She was like, ‘Oh, no, that’s normal because you just had a baby and then you put the birth control in.’ I said, ‘You never told me that.’” [Black and Hispanic participant aged 19 years)  ● “When I first got pregnant, they wouldn’t tell me about that stuff. Then when I was around my 30 week, they were talking about what type of birth control I wanted to take, and I told them the implant. I guess they just wanted to prevent me from getting pregnant again, so they would tell me a lot of stuff, what to do to not get pregnant again. It was kind of pushy because I guess they wanted me to take birth control. They were asking me which one I wanted to take and stuff like that.” [Hispanic participant aged 18 years]  ● “They was making it seem like it was the worst thing in the world to get because we was talking about the IUDs and the implant and they was trying to measure the both of them and trying to...I don’t know. He was trying to convince me to get the implant. It was kind of pushy.” [Black and Hispanic participant aged 17 years] |
