# Supplementary figures and images for: Cocreating First Steps, a Toolkit to Improve Adolescent Sexual and Reproductive Health Services: Qualitative Human-Centered Design Study With Hispanic and Black Adolescent Mothers in New York City
Source: JMIR Pediatr Parent. 2024 Nov 19;7:e60692. doi: 10.2196/60692 (PMC11615555; doi:10.2196/60692)

**Supplemental material:**

Personas, scenarios, and brainstorming


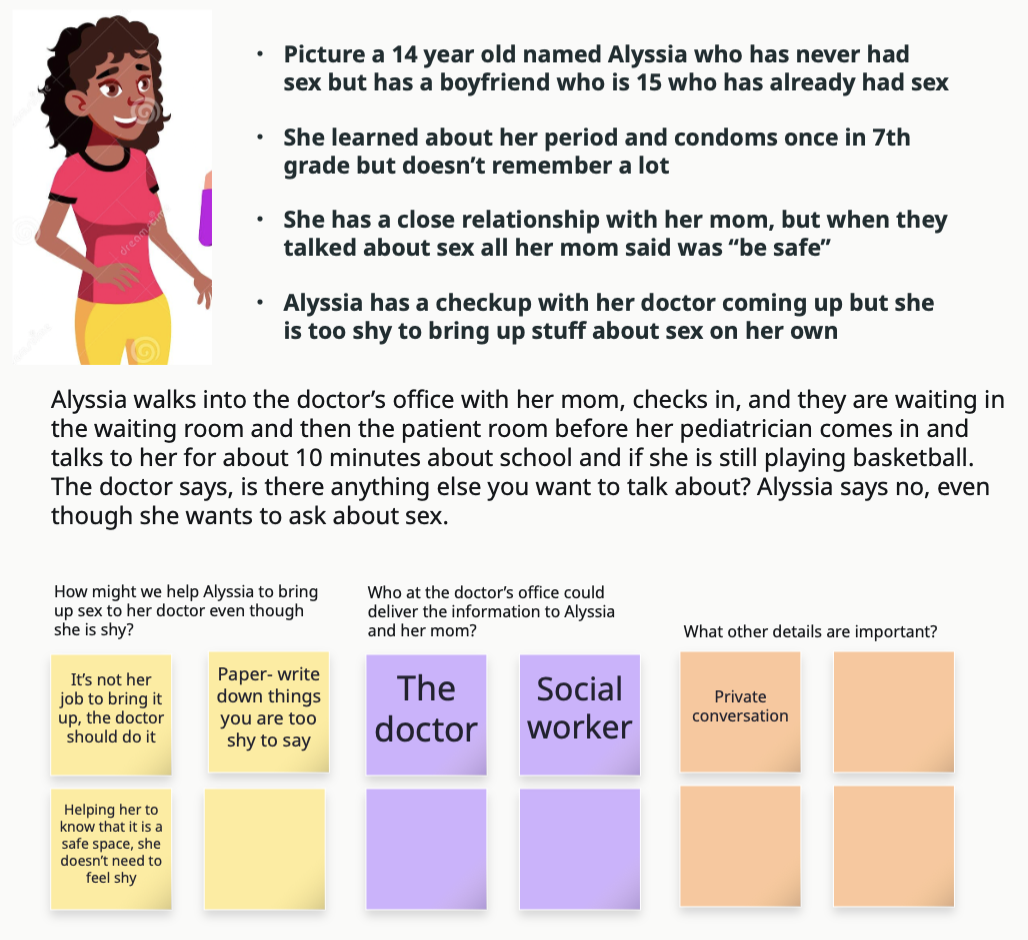


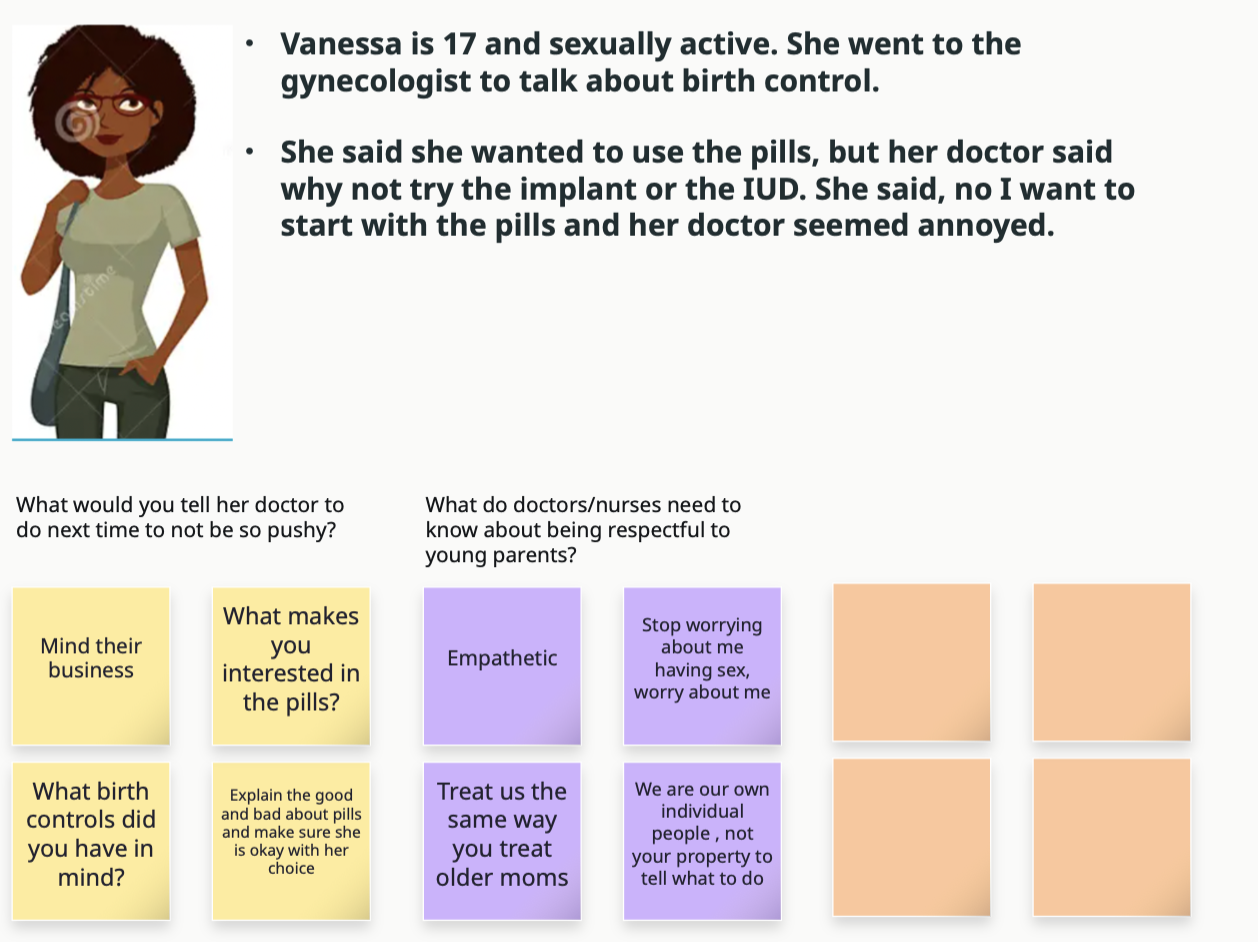

Supplement: Multimedia Appendix 2 [file pediatrics_v7i1e60692_app2.docx]
